# High Auxin and High Phosphate Impact on RSL2 Expression and ROS-Homeostasis Linked to Root Hair Growth in *Arabidopsis thaliana*

**DOI:** 10.3389/fpls.2018.01164

**Published:** 2018-08-14

**Authors:** Silvina Mangano, Silvina P. Denita-Juarez, Eliana Marzol, Cecilia Borassi, José M. Estevez

**Affiliations:** Fundación Instituto Leloir and Instituto de Investigaciones Bioquímicas de Buenos Aires (IIBBA-CONICET), Buenos Aires, Argentina

**Keywords:** Arabidopsis thaliana, auxin, NADPH oxidases, peroxidases, phosphate, root hairs, ROS

## Abstract

Root hair size determines the surface area/volume ratio of the whole roots exposed to the nutrient and water pools, thereby likely impacting nutrient and water uptake rates. The speed at which they grow is determined both by cell-intrinsic factors like hormones (e.g., auxin) and external environmental signals like nutrient availability in the soil (e.g., phosphate). Overall root hair growth is controlled by the transcription factors RSL4 and RSL2. While high levels of auxin promote root hair growth, high levels of inorganic phosphate (Pi) in the media are able to strongly repress RSL4 and RSL2 expression linked to a decreased polar growth. In this work, we inquired the mechanism used by root hairs to integrate conflicting growth signals like the repressive signal of high Pi levels and a concomitant high auxin exposure that promotes growth and questioned whether these complex signals might activate known molecular players in root hair polar growth. Under these conditions, RSL2 expression (but not RSL4) is activated linked to ROS production and root hair growth. On the other hand, by blocking ROS production derived from the NADPH Oxidase C (or RBOHC for RESPIRATORY BURST OXIDASE HOMOLOG C) and ROS production from Secreted type-III Peroxidases (PERs), it was possible to repress the auxin growth-promoting effect. This study identifies a new layer of complexity between auxin, Pi nutrient availability and RSL2/RSL4 transcription factors all acting on ROS homeostasis and growth at the root hair level.

## Introduction

How plant cells regulate their size is one of the most fascinating questions in current plant biology. Root hairs are single plant cells that can expand several hundred-fold their original size and they are an excellent model system for learning about cell size regulation. Root hair size determines the surface area/volume ratio of the whole roots exposed to the nutrient and water pools, thereby likely impacting nutrient and water uptake rates. Their growth speed is determined by cell-intrinsic factors like hormones (e.g., auxin and ethylene) and external environmental signals like nutrient availability in the soil (e.g., phosphate and nitrates) (Lee and Cho, 2013; Zhang et al., 2016; Feng et al., 2017; Franciosini et al., 2017; Mangano et al., 2017).

The root hair polar growth program is initially triggered by the transcription factors (TFs) of basic helix-loop-helix (bHLH) family RHD6 (ROOT HAIR DEFECTIVE 6) /RSL1 (ROOT HAIR DEFECTIVE 6 LIKE 1) in the initiation phase, and then, activated by the expression of RSL4/RSL2 during the elongation phase (Menand et al., 2007; Yi et al., 2010). Hormonal and environmental cues converge to regulate the expression of RSL4, which controls the final root hair cell size (Yi et al., 2010; Datta et al., 2015; Song et al., 2016; Franciosini et al., 2017; Rymen et al., 2017). In this process, however, other TFs might act in a coordinated manner under specific signals such as Pi starvation and ethylene for EIN3 (ETHYLENE INSENSITIVE 3)/EIL1 (ETHYLENE INSENSITIVE 3-LIKE 1) and LRL3 (LOTUS JAPONICA ROOTHAIRLESS-LIKE1) (Yi et al., 2010; Song et al., 2016; Feng et al., 2017). On the other hand, recently it was found that the trihelix factor GT-2-LIKE (GTL1) and its homolog DF1 repress root hair growth by negatively regulating the expression of RSL4 (Shibata et al., 2018). Recently, it was found that, by releasing several ARFs (e.g., ARF5, ARF7, ARF8, and ARF19) from Aux/IAA proteins, auxin directly activates RSL4 expression and controls root hair growth linked to ROS-homeostasis involving three RESPIRATORY BURST OXIDASE HOMOLOG proteins (e.g., RBOHC,H,J) and four type-III secreted peroxidases (e.g., PER1,60,44,73) (Mangano et al., 2017). In addition, RSL2 was also involved in the auxin-mediated growth response although its mode of action at the molecular level was unclear (Mangano et al., 2017). Conversely, high levels of inorganic phosphate (Pi) in the soil (or in the media) are able to strongly repress RSL4 expression linked to polar growth by an unknown mechanism while Pi-starvation results in an extensive outgrowth of root hairs. This response is associated with increased activity of two MYB-transcription factors, PHR1 (PHOSPHATE STARVATION RESPONSE) and PHR1-LIKE1 (PHL1), the homeodomain transcription factor AL6/PER2 (ALFIN-LIKE6/Pi DEFICIENCY ROOT HAIR DEFECTIVE 2), EIN3/ENL1, and RSL4 (Bustos et al., 2010; Yi et al., 2010; Chandrika et al., 2013; Datta et al., 2015; Song et al., 2016; Feng et al., 2017; Franciosini et al., 2017). Recently, it was shown that low Pi induces root hair growth by stimulating high auxin synthesis in the root cap, auxin transport possible mediated by AUX1, and ARF19 activation of RSL2 and RSL4 expression (Bhosale et al., 2018). Although the molecular mechanisms by which either auxin or Pi controls root hair growth are partially known (Song et al., 2016; Feng et al., 2017; Mangano et al., 2017; Bhosale et al., 2018), much less is understood for complex signals acting at the same time. Here, we inquired the mechanism by which root hairs integrate conflicting growth signals like the repressive high Pi level cue and a concomitant high auxin exposure that should promote growth and questioned whether these complex signals might activate known molecular players in polar growth. Surprisingly, under high-Pi and high auxin RSL2 but not RSL4 plays an important role on ROS homeostasis and root hair growth indicating that not only auxin is responsible for this growth response but there is a direct effect of high Pi on the transcriptional regulation of RSL2.

## Materials and Methods

### Plant Growth and Mutant Isolation

*Arabidopsis thaliana* Columbia-0 (Col-0) was used as the wild type (Wt) genotype in all experiments, unless otherwise stated. All mutants and transgenic lines tested are in this ecotype. Seeds were germinated on agar plates in a Percival incubator at 22°C in a growth room with 16 h light/8 h dark cycles for 10 days at 140 μmol m^-2^s^-1^ light intensity. Plants were transferred to soil for growth under the same conditions as previously described at 22°C. For identification of T-DNA knockout lines, genomic DNA was extracted from rosette leaves. Confirmation by PCR of a single and multiple T-DNA insertions in the target RBOH and PER genes were performed using an insertion-specific LBb1 or LBb1.3 (for SALK lines) or Lb3 (for SAIL lines) primer in addition to one gene-specific primer. To ensure gene disruptions, PCR was also run using two gene-specific primers, expecting bands corresponding to fragments larger than in Wt. In this way, we isolated homozygous lines (for all the genes mentioned above). Mutants list is detailed in **Supplementary Table S1**.

### Growth Media. RBOHs Inhibition and Auxin Treatment

Sterilized seeds were stored at 4°C in sterile water for 48 h and then were germinated on agar plates containing modified Hoagland solution that contained 1 mM Ca(NO_3_)_2_, 50 μM CaCl_2_, 0.25 mM MgSO_4_, 50 μM Fe-NaEDTA, 1 mM KCl, 2 μM MnSO_2_⋅2H_2_O, 0.5 μM CuSO_4_⋅5H_2_O, 0.5 μM Na_2_MoO_4_⋅2H_2_O, 2 μM Zn SO_4_⋅7H_2_O and 2.5 mM MES containing low phosphate [5 μM (NH_4_)_2_HPO_4_]. Media were adjusted to a pH of 5.7 and solidified using 0.8% agar (Duchefa). After 5 days plants were transferred into modified Hoagland solution with low phosphate [5 μM (NH_4_)2HPO_4_] or high phosphate [5 mM (NH_4_)_2_HPO_4_]. Low and high phosphate were combined with 100 nM of indole-3-acetic acid (IAA) or/and 15 μM of VAS2870 [VAS, for 3-Benzyl-7-(2-benzoxazolyl)thio-1,2,3-triazolo(4,5-d)pyrimidine] or/and 100 μM SHAM (salicylhydroxamic acid). After 5 days (10 days old), quantitative analysis of root hair phenotypes and total ROS measurements with H_2_DCF-DA were made.

### Root Hair Phenotype

For quantitative analysis of root hair phenotypes in *rboh* mutants and Wt Col-0, 200 fully elongated root hairs were measured (n roots = 30) from seedlings grown on vertical plates for 10 days. Values are reported as the mean ± SD using the Image J 1.50b software. Measurements were made on images captured with an Olympus SZX7 Zoom microscope equipped with a Q-Colors digital camera.

### H_2_DCF-DA Probe Used to Measure Total ROS

*A. thaliana* seedlings were grown in sterile plates with agar 1% for 8 days in chamber at 25°C with a continuous light. These seedlings were incubated in darkness in a slide for 10 min with the 2^′^,7^′^-Dichlorodihydrofluorescein diacetate (H_2_DCF-DA) 50 μM at room temperature. Samples were observed with a confocal microscope equipped with 488 nm argon laser and BA510IF filter sets. A 10X objective; 0.30 NA; 4.7 laser intensity; 1.1 off set; 440 PMT (for highest ROS levels) 480 PMT (for ROS media) and 3 gain were used. Images were taken scanning XZY with a 2 um between focal planes. Images were analyzed using ImageJ. To measure ROS highest levels, a circular ROI (*r* = 2.5) was taken in the zone of the root hair with highest intensities. To measure ROS mean, the total area of the root hair was taken. Pharmacological treatments were carried out with a combination of the following reagents: 1–20 mM Pi, 100 nM IAA, and 15 μM of VAS2870. We washed the sample with a MS 0.5x solution and the image acquisition was made with a 10X objective and 400 ms of exposure time in an epifluorescence microscope (Zeiss, Imager A2). To measure ROS levels a circular ROI (*r* = 2.5) was taken in the tip of the root hair. Values are reported as the mean ± SD using the Image J 1.50b software.

### HyPer Sensor to Measure _cyt_H_2_O_2_

HyPer consists of a circularly permuted YFP (cpYFP) molecule coupled to a regulatory domain of the *Escherichia coli* H_2_O_2_ sensor OxyR (1–4). When exposed to H_2_O_2_, the excitation peak of cpYFP shifts from 420 to 500 nm, while the emission peak remains at 516 nm allowing it to be used as a ratiometric biosensor (Mangano et al., 2017). Ten day-old *A. thaliana* seedlings expressing the fluorescent HyPer biosensor were used. Root hairs were ratio imaged with the Zeiss LSM 510 laser scanning confocal microscope (Carl Zeiss) using a 40× oil-immersion objective, 1.2 numerical aperture. The HyPer biosensor was excited with both the 405 nm blue diode laser and with the 488 nm argon laser. The emission (516 nm) was collected using a primary dichroic mirror and the Meta-detector of the microscope. For time-lapse analysis, images were collected every 3 s. To measure ROS highest levels, a circular ROI (*r* = 2.5) was taken in the root hair tip for every image of the time lapse. Treatments were made *in vivo* with 5 mM Pi, 100 nM IAA. Values are reported as the mean ± SD using the Image J 1.50b software.

### Quantitative Reverse Transcriptase PCR (qRT-PCR)

Total RNA was isolated from 10-day-old seedling roots (40 for each line) using the RNAzol RT (MRC). cDNA was synthesized using M-MLV Reverse Transcriptase (Promega). qRT-PCR analyses were performed using LightCycler480 SYBR Green I Master-Roche. Gene-specific signals were normalized relatively to PP2A (AT1G69960; serine/threonine protein phosphatase 2A) signals. Each qRT-PCR reaction was performed in triplicate, and each experiment was repeated three times using independent preparations of RNA. Primers used are listed in **Supplementary Table S2**.

## Results

### Auxin Overcomes Pi-Repression of Root Hair Growth in a ROS-Dependent Manner

**FIGURE 1:**
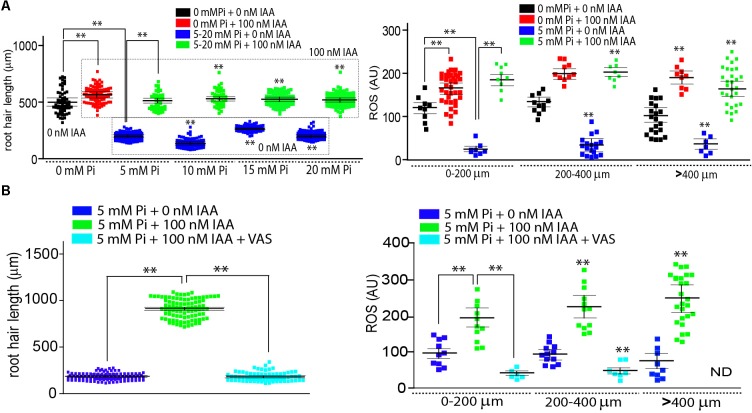
Auxin overcomes high Pi root hair growth repression by enhancing ROS production. **(A)** Auxin circumvents root hair growth repression imposed by increased levels of Pi by enhancing ROS production. Root hair length (mean ± SD) was measured in Wt Col-0 roots non-treated, treated with 100 nM IAA (indole 3-acetic acid) or with 5 mM inorganic Pi, or with both treatments at the same time (on the left). Total ROS levels generated by oxidation of H_2_DCF-DA were measured at the root hair tip in different stages of root hair development in the four different treatments (on the right). **(B)** Auxin recovery of root hair growth is dependent on ROS production. Root hairs treated with 100 nM IAA in the presence of high levels of Pi and VAS inhibitor fail to recover their normal growth (on the left). ROS levels were downregulated in the root hairs treated with 100 nM IAA in the presence of high levels of Pi and VAS inhibitor (on the right). ND, not detected. *P* value of one-way ANOVA, (^∗∗^) *P* > 0.001, (^∗^) *P* > 0.01. NS = not significantly different. Error bars indicate ± SD from 3 biological replicates. AU = Arbitrary Units.

To study how conflicting signals are integrated during polarized growth of root hair, we tested a growth repressive condition with high Pi levels and a concomitant high auxin exposure that should promote growth (**Figure 1A**). Increased levels of Pi (5 mM or higher) in the media (using modified Hoagland solution) were able to strongly repress root hair growth as previously reported although at lower concentrations (Datta et al., 2015; Song et al., 2016) as well as to greatly reduce the reactive oxygen species (ROS) levels in root hair cells in the *A. thaliana* model (**Figure 1A**). ROS levels were measured at the tip of root hair cells with the cell permeable probe H_2_DCF-DA (2^′^,7^′^-dichlorodihydrofluorescein diacetate) that becomes irreversibly fluorescent under ROS-oxidation. An exogenous auxin supply (100 nM IAA, Indole 3-Acetic Acid) was able to restore root hair growth independently of the level of Pi in the media (**Figure 1A**). In addition, while the presence of Pi in the medium affects ROS production in the root hair, ROS levels were higher in the auxin-treated root hair in contrast with the no treated even in the presence of high levels of Pi, suggesting that both signals, high Pi levels as well as high auxin, operated in an opposite manner (**Figure 1A**). We did not decided any inhibition in root hair growth at 1 mM Pi so we decide to continue to work with 5 mM as a high Pi condition.

**FIGURE 2:**
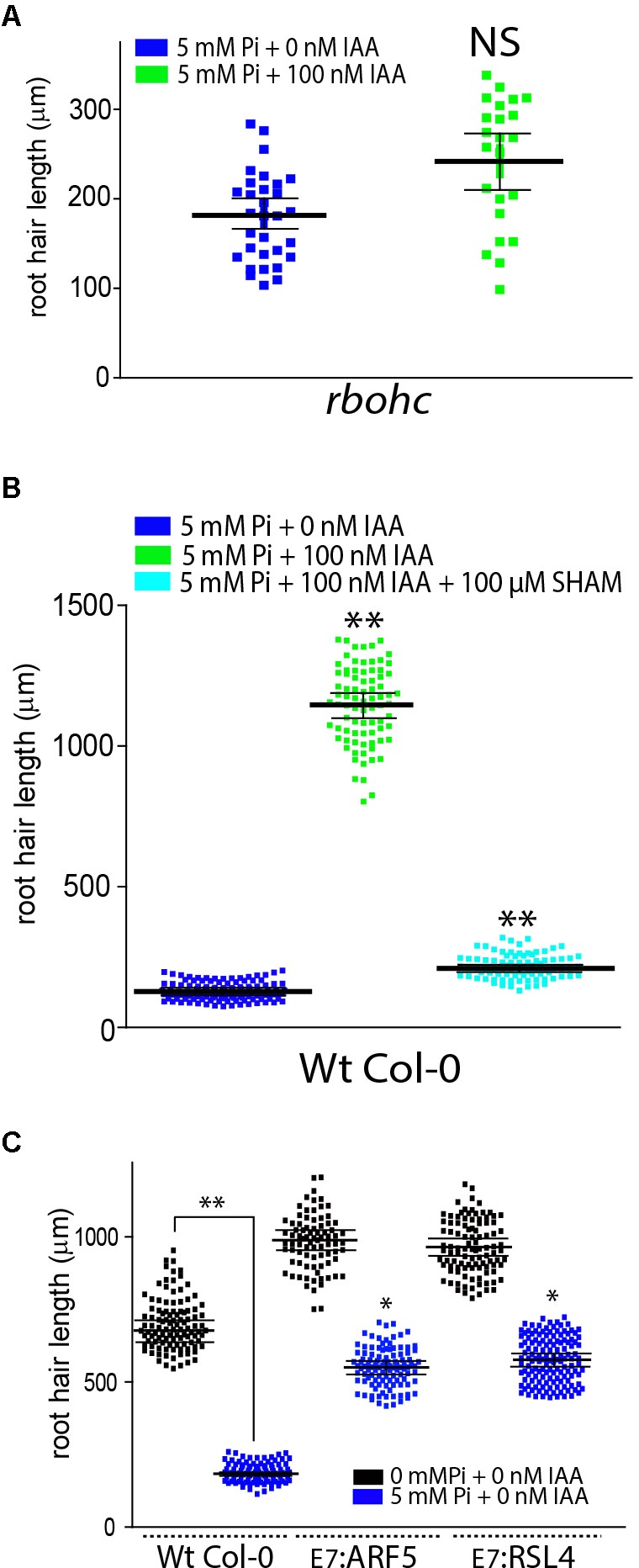
Auxin fails to overcome high Pi root hair growth repression in a ROS-depleted background. **(A)** Auxin fails to circumvent root hair growth repression imposed by increased levels of Pi in a ROS-depleted *noxc* mutant. Root hair length (mean ± SD) was measured in *noxc* roots treated with 5 mM inorganic Pi with or without 100 nM IAA (indole 3-acetic acid) (on the left). Total ROS levels generated by oxidation of H_2_DCF-DA were measured at the root hair tip in different stages of root hair development in the four different treatments (on the right). **(B)** Auxin fails to rescue root hair growth repression imposed by increased levels of Pi in a ROS-depleted PER environment by the inhibition of SHAM (salicylhydroxamic acid). Root hair length (mean ± SD) was measured in roots in the presence of SHAM and treated with 5 mM inorganic Pi with or without 100 nM IAA (indole 3-acetic acid) (on the left). Total ROS levels generated by oxidation of H_2_DCF-DA were measured at the root hair tip in different stages of root hair development in the four different treatments (on the right). **(C)** High levels of ARF5 and RSL4 expression in root hair cells (EXPANSIN 7 promoter, E7) are able to partially overcome the growth repression imposed by high levels of Pi on cell elongation. Comparisons are made between Wt Col-0 and _E7_:ARF5 or _E7_:RSL4 lines under high levels of Pi. *P* value of one-way ANOVA, (^∗∗^) *P* > 0.001, (^∗^) *P* > 0.01. NS = not significantly different. Error bars indicate ± SD from 3 biological replicates.

Then, it was tested whether the suppression of ROS derived from RBOHs activities with VAS2870 (VAS, a specific RBOHs inhibitor) might affect auxin growth-effect in the presence of Pi (**Figure 1B**). VAS treatment was able to revert the auxin growth-effect at the root hair level by down-regulating RBOHs-derived ROS production (**Figure 1B**). A similar repressive effect was obtained when *noxc* mutant with low-ROS, was incubated with auxin in the presence of high Pi-levels (**Figure 2A**). Inhibiting ROS-derived from PERs with SHAM (salicylhydroxamic acid) also blocked the auxin effect (**Figure 2B**). This confirms that, even in Pi presence, drastic changes in ROS homeostasis controlled mostly by RBOHC and by several PERs (Mangano et al., 2017) inhibit auxin growth-promoting effect and that high Pi levels as well as high auxin worked in the opposite way by a ROS-dependent mechanism. In addition, over-expression of either RSL4 or Auxin Response Factor 5 (ARF5) under the control of the root hair EXPANSIN 7 promoter (E7) that previously showed enhanced root hair growth and high-levels of ROS (Mangano et al., 2017) were much more insensitive to high Pi levels that Wt Col-0 and they developed almost regular extended root hairs (**Figure 2C**). This result indicates that high expression of RSL4 or constitutively activated auxin signaling, both are able to partially repress the growth-effect of high levels of Pi.

**FIGURE 3:**
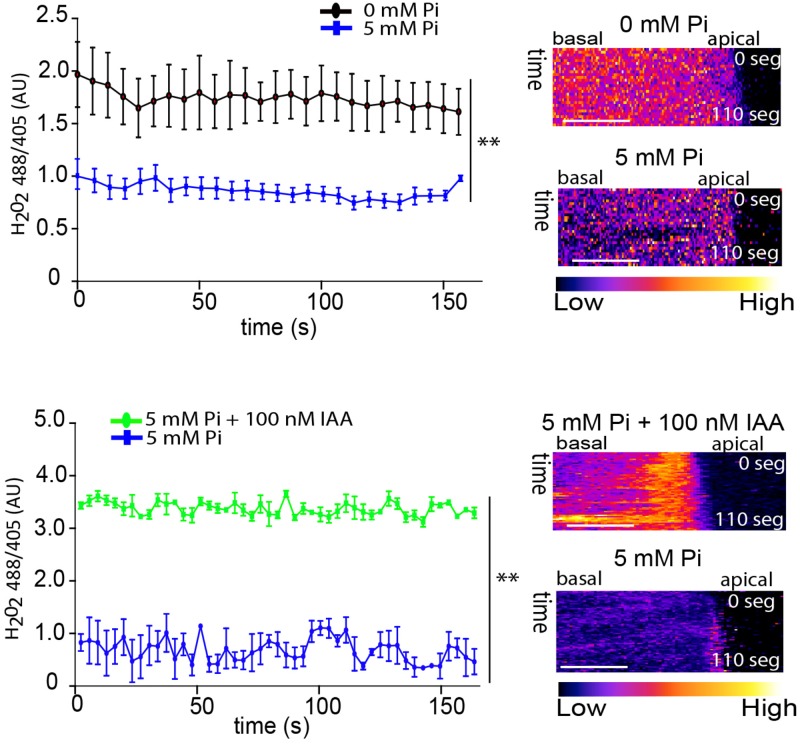
Auxin triggers changes in _cyt_H_2_O_2_ levels detected with HyPer under high Pi levels. _cyt_H_2_O_2_ levels Wt Col-0 root hairs expressing HyPer sensor treated with 5 mM Pi and 100 nM IAA. _cyt_H_2_O_2_ levels are based on the ratio 488/405 nm of HyPer biosensor at the root hair tip over 200 s. On the right, selected kymographs resulting of this analysis, only for root hairs of >200 μm in length. Scale bar = 5 μm. *P* value of one-way ANOVA, (^∗∗^) *P* > 0.001, (^∗^) *P* > 0.01. NS = not significantly different. Error bars indicate ± SD from 3 biological replicates. AU = Arbitrary Units.

To corroborate the ROS measurements performed with the H_2_DCF-DA probe as well as to determine whether the main ROS molecule involved was hydrogen peroxide (H_2_O_2_), we measured cytoplasmic H_2_O_2_ (_cyt_H_2_O_2_) levels using a genetically encoded YFP-based H_2_O_2_ sensor, HyPer (Mangano et al., 2017). Results obtained were similar to previous ones, since high levels of Pi repressed cytoplasmic H_2_O_2,_ while exogenous auxin was able to trigger an up-regulation of the ROS signal in the presence of high Pi in root hairs (**Figure 3**). Altogether, these results indicate that auxin is able to overcome Pi growth repression by restoring the ROS production, which is required for trigger proper polar-growth.

### Expression of RSL2 Is Required to Bypass Pi Growth Repression in the Presence of Auxin

**FIGURE 4:**
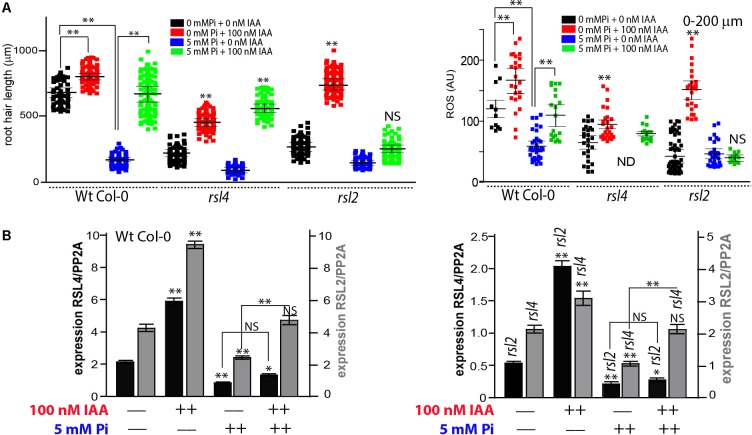
RSL2 but not RSL4 mediates the auxin mediated growth recovery response in the presence of high Pi levels. **(A)** Root hair length (mean ± SD) was measured in Wt Col-0, *rsl4* and *rsl2* roots non-treated, treated with 100 nM IAA (indole 3-acetic acid) or with 5 mM inorganic Pi, or with both treatments at the same time. ROS levels were partially recovered in Wt but not in *rsl2* when treated with 100 nM IAA in the presence of high levels of Pi. No ROS was detected in *rsl4* under high levels of Pi. Only root hairs <200 μm in length were analyzed. **(B)** Under high levels of Pi, auxin triggers the expression of both RSL4 and RSL2. Levels of RSL4 and RSL2 expression in Wt Col-0 roots non-treated, treated with 100 nM IAA (indole 3-acetic acid) or with 5 mM inorganic Pi, or with both treatments at the same time (on the left). Levels of RSL4 and RSL2 expression in *rsl2* and *rsl4*, respectively, non-treated, treated with 100 nM IAA (indole 3-acetic acid) or with 5 mM inorganic Pi, or with both treatments at the same time (on the left). To simplify the graphs, Col 0 and the *rsl2-* and *rsl4-* mutants are shown separately although the experiments were performed at the same time. Each experiment, with the three genotypes together (Wt Col-0, *rsl2-* and *rsl4-*) was performed in triplicate, and qRT-PCR reaction was repeated three times using independent preparations of RNA. Comparisons are made for the same genetic background between levels of gene expression (of RSL2 or RSL4) in non-treated samples (no IAA/no Pi) versus treated with IAA or Pi. In addition, Pi-treated samples are compared only to Pi+IAA-treated roots. *P*-value of one-way ANOVA, (^∗∗^) P > 0.001, (^∗^) P > 0.01. NS = not significantly different. Error bars indicate ± SD from 3 biological replicates. AU = Arbitrary Units.

In order to gain insights into the molecular mechanism behind these high Pi-auxin conflicting responses, we tested root hair growth linked to ROS levels in the absence of RSL2 and RSL4 TFs that were shown to regulate the auxin-mediated root hair growth response (Datta et al., 2015; Mangano et al., 2017). The double mutant *rsl2 rsl4* is not capable of developing visible root hairs in any condition tested, neither under high levels of auxin nor ethylene nor nutrient deprivation, suggesting that both TFs arenecessary to run the basic transcriptional machinery to produce root hairs (Feng et al., 2017; Mangano et al., 2017). A high Pi level strongly represses the expression of both RSL2 and RSL4 TFs in the Wt Col-0 roots while auxin treatment up-regulates both TFs and not only RSL4, as previously indicated (Pires et al., 2013). When *rsl4* (that lacks RSL4 transcripts) was tested, showed that in both conditions, in the absence or presence of Pi, the root hair length increases with the addition of auxins, a similar response to that observed in Wt, indicating that auxin can rescue the negative effect of high-Pi levels even in absence of *rsl4*, possibly mediated by RSL2 action. In agreement with this idea, when RSL2 was lacking (in *rsl2* mutant), the addition of auxin in a medium containing 5 mM Pi does not produce any effect on root hair length although this mutant is able to respond to the induction of this hormone in the absence of Pi (**Figure 4A**). This indicates that RSL2 mediates the auxin rescue response in the presence of high Pi levels through an independent RSL4 mechanism. In addition, ROS measurements in Wt Col-0 as well as in *rsl4* and *rsl2* mutants positively correlate with growth responses in all conditions tested (**Figure 4A**) confirming the connexion that exists between auxins, Pi, ROS production and polarized root hair growth.

Then, levels of *rsl2* and *rsl4* transcripts were measured in their respective mutant backgrounds (*rsl4* and *rsl2*, respectively) to study whether these genes are regulated at the transcriptional level and to define if any of these two TFs depends on the presence of the other to be expressed under these conflicting growth conditions (**Figure 4B**). In Wt Col-0 we observe that, in the absence of Pi, the addition of auxins produces an increase in the accumulation of transcripts of both genes. However, in the presence of Pi we only observed an auxin positive regulation of *rsl2* expression but not linked to *rsl4* expression. On the other hand, in the *rsl4* mutant background, the expression of *rsl2* was down-regulated to ∼50% of the levels found in Wt Col-0, while in the *rsl2* background the levels of *rsl4* expression were three to four-times lower in all conditions measured (e.g., no Pi or auxin added, high Pi level, high auxin, or both). This indicates that the absence of *rsl2* might affect *rsl4* expression, and *rsl2* transcripts also went down when *rsl4* was lacking, suggesting a positive feed-forward loop between RSL2 and RSL4 by a direct or indirect mechanism that requires further investigation.

## Discussion

**FIGURE 5:**
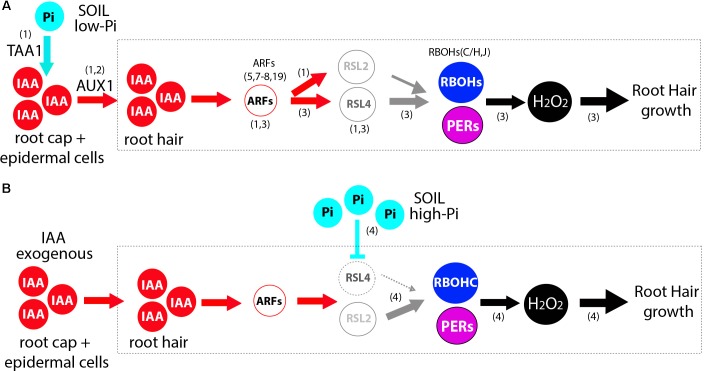
Proposed models of auxin-RSL2/RSL4 regulation of ROS-mediated polar root hair growth in the absence and in the presence of high Pi. Transcriptional responses under high level of auxin in the absence of Pi (A) and in the presence of high Pi together with high levels of auxin are shown. This proposed model is based on evidences shown in (1) Bhosale et al., 2018; (2) Giri et al., 2018; (3) Mangano et al. (2017); (4) current work. **(A)** Low levels of Pi in the soil (media) triggers auxin biosynthesis (involving TAA1) and transport (by AUX1) from the root cap and epidermis cells to the root hair cells. High levels of auxin then induces the activation of several Auxin Response Factors (ARFs; ARF19 and ARF5,7,8). The bHLH transcription factor RSL4 as well as RSL2 are both transcriptionally activated by high levels of auxin (IAA) and its expression is directly regulated by these ARFs. Through RSL4 and possibly RSL2, auxin activates the expression of two RBOHs (RBOHC,J) and four PERs (PER1,44,60,73) that together regulate ROS homeostasis in the apoplast (in combination with RBOHH). **(B)** High levels of Pi in the presence of high levels of auxin are able to activate the expression of RSL2 and control ROS homeostasis derived from RBOHs and PERs activities. Solid lines indicate transcriptional activation or metabolite production. The main reactive oxygen species (ROS) highlighted is hydrogen peroxide (H_2_O_2_).

A general model is proposed to understand how auxin together with high Pi levels controls root hair growth based on this current knowledge by integrating our results together with previous findings (Mangano et al., 2017; Bhosale et al., 2018; Giri et al., 2018) (**Figure 5**). Since high levels of Pi strongly repress root hair growth and ROS production, Pi might operate at several regulatory points such as down-regulating auxin biosynthesis, auxin conjugation and transport within the roots toward the trichoblast cells (Velasquez et al., 2016), and, then, indirectly affecting RSL4/RSL2 expression together with downstream target genes. This scenario is compatible with a recent discovery where low Pi induces auxin biosynthesis acting on TAA1 gene (for *TRYPTOPHAN AMINOTRANSFERASE OF ARABIDOPSIS1*) in the root cap and epidermal cells, auxin transport by AUX1 (In *A. thaliana* and Rice) and ARF19 expression linked to RSL2 and RSL4 mediated root hair growth (Bhosale et al., 2018; Giri et al., 2018). Surprisingly, under high Pi together with high auxin levels, we have found that is RSL2 but not RSL4 root hair growth. This suggests that, in the presence of auxin, high Pi triggers some changes in RSL2-RSL4 activation levels that stimulate the growth of root hair not detected before (Mangano et al., 2017; Bhosale et al., 2018). It is plausible that RSL2 and RSL4 would be able to form protein dimers as reported for several other TFs that contain a HLH domain (Feller et al., 2011), more precisely heterodimers (e.g., RSL2-RSL4) and homodimers (e.g., RSL2-RSL2 and RSL4-RSL4) with slightly different downstream transcriptional target genes depending on the hormonal and environmental conditions. In the same manner as RSL4 is able to self-activate by a forward positive-loop (Hwang et al., 2017), we hypothesize that RSL2 would be also capable of doing the same. In addition, RSL2 highly active in the elongation root zone would be able to up-regulate RSL4 activation in the already elongated zone based on the expression pattern shown recently (Bhosale et al., 2018), but not vice versa where RSL4 activates RSL2, as it was suggested in a previous work (Pires et al., 2013). Further studies are now required to establish the interconnections between the two TFs under the different environmental and hormonal conditions. Since RSL4 is directly activated by ARF5 (and possibly by several other ARFs including ARF7,8,19) in trichoblast cells under high auxin (Mangano et al., 2017), we also postulate that RSL2 would be up-regulated by some of these ARFs, since auxin increases its expression by two and a half times (**Figure 4**). In agreement, several consensus Aux-RE sites are found in the regulatory region of RSL2 as possible targets for ARFs binding (Bhosale et al., 2018). In addition, recently it was shown that ARF19 might direct the expression of RSL2 and RSL4 under low-Pi condition (Bhosale et al., 2018) although it is unclear how ARF19 that is active in the elongation and differentiation roots zones, is possibly able to first trigger RSL2 and latter on in root development activate RSL4 expression. Overall, how the fine-tune regulation of ARFs on the RSL2-RSL4 expression is coordinated under auxin and Pi variable conditions still remains to be discovered. In addition, we found that root hair growth under this condition (Pi + auxin) requires ROS production derived from RBOHC as well as from a pool of PERs in a similar manner than when Pi is present in low levels (Mangano et al., 2017) suggesting that several RSL4 and RSL2 downstream targets might be similar in both conditions. Multiple hormonal signals other than auxin may operate in these cells since for example abscisic acid (ABA) was able to repress RSL2 (and RSL3) expression through the TF OBP4 (OBF BINDING PROTEIN 4) and negatively regulate root hair growth (Rymen et al., 2017). In summary, although it has been previously shown that auxin triggers root hair cell elongation while high Pi levels repress its growth, how both conflicting signals might act together was unknown. This study identifies a new layer of complexity between RSL2/RSL4 TFs acting on ROS homeostasis under conflicting growth signals at the root hair level.

## Author Contributions

SM performed all the experiments, reviewed the text, figures, and references. SD-J performed some of the HyPer experiments. EM and CB reviewed the text, references, and figures. JE conceived the project, designed the figures, and wrote the article with contributions from all the authors.

## Conflict of Interest Statement

The authors declare that the research was conducted in the absence of any commercial or financial relationships that could be construed as a potential conflict of interest. The reviewer JS and handling Editor declared their shared affiliation.
