# Supplementary material for: High Auxin and High Phosphate Impact on RSL2 Expression and ROS-Homeostasis Linked to Root Hair Growth in Arabidopsis thaliana
Source: Front Plant Sci. 2018 Aug 14;9:1164. doi: 10.3389/fpls.2018.01164 (PMC6102359; doi:10.3389/fpls.2018.01164)
Supplement: Supplementary file 1 [file Table_1.pdf]

Supplementary Table S1. RSLs and NADPH oxidase C (RBOHC) mutant lines used in this study. All are in Col-0 background.

Name	Locus	Mutant name/transgenic line	Mutant code	References
		**ARF5/RSL4**		
**E7:ARF5**	At1g19850	Overexpressor of ARF5	-	Mangano et al. (2017)
**E7:RSL4**	At1g27740	Overexpressor of RSL4	-	Mangano et al. (2017)
		**RSLs**		
**RSL2**	At3g33880	*rsl2-1*	SAIL line 514C04	Yi, K. et al. (2010)
**RSL4**	At1g27740	*rsl4-1*	GT_5_105706	Yi, K. et al. (2010)
		**RBOHs**		
**RBOHC**	At5g51060	*rbohc-1*	Salk_071801	Lee, Y. et al. (2013)

Supplementary Table S2. List of primers used for qPCR.

Gene	Position from start codon	Sequence	Direction
**RSL2**	896	CCCCAATGGAACAAAGGTC	Forward
At3g33880	1036	TCTCGGTGAGCTGAGACCAA	Reverse
**RSL4**	597	GTGCCAAACGGGACAAAAGT	Forward
At1g27740	735	TTGTGATGGAACCCCATGTC	Reverse
**PP2A**	1785	TAACGTGGCCAAAATGATGC	Forward
At1g69960	1845	GTTCTCCACAACCGCTTGGT	Reverse
